# A general-purpose approach to multi-agent Bayesian optimization across decomposition methods

**DOI:** 10.1007/s11081-024-09953-w

**Published:** 2025-01-20

**Authors:** Dinesh Krishnamoorthy

**Affiliations:** Department of Mechanical Engineering, Eindhoven University of Technology, 5600 MB Eindhoven, The Netherlands

**Keywords:** Bayesian optimization, Distributed optimization, Multi-agent systems

## Abstract

This paper proposes a general-purpose multi-agent Bayesian optimization (MABO) where agents are connected via shared variables or constraints, and each agent’s local cost is unknown. The proposed approach is general-purpose in the sense that it can be used with a broad class of decomposition methods, whereby we augment traditional BO acquisition functions with suitably derived coordinating terms to facilitate coordination among subsystems without sharing local data. Regret analysis is also carried out for the general-purpose MABO framework, which reveals that the cumulative regret of the proposed general-purpose MABO is the sum of individual regrets and is independent of the coordinating terms. This adaptability to different decomposition methods ensures versatility across diverse distributed optimization scenarios. Numerical experiments validate the effectiveness of the proposed MABO framework for different classes of decomposition methods.

## Introduction

Multi-agent decision-making problems, formulated as optimization problems, arise in a wide range of application areas, where a collection of local decision-making agents collaborate to achieve a common goal. Examples include networked systems like sensor networks and communication networks, where nodes need to collaborate to optimize network performance or resource allocation. In transportation systems, such as vehicle platooning or traffic control, individual vehicles must coordinate their actions to improve overall system efficiency. In power systems, distributed energy resources must coordinate their operation to ensure grid stability and reliability. In these examples, distributed optimization techniques play a crucial role, where each agent solves its local optimization problem while coordinating with the other agents to achieve the overall system optimum (Lasdon 2002). The coordination may be in the form of decentralized peer-to-peer coordination or facilitated by a central coordinator.

Typically, the local optimization problem requires detailed models/analytical expressions that relate the local decision variables to the local objective function. However, obtaining accurate and reliable models is a time-consuming and tedious task in many engineering applications, especially in complex and next generation systems with limited domain knowledge. The modeling effort is one of the major bottleneck for traditional distributed optimization, especially for applications where reliable models are not readily available. Black-box optimizations methods that do not require any models is a promising alternative to circumvent this challenge.

Bayesian optimization (BO) is a powerful optimization framework that has gained popularity in recent years due to its effectiveness in optimizing black-box functions with noisy evaluations. At its core, BO utilizes probabilistic surrogate models, typically Gaussian processes (GPs), to model the unknown objective function. By iteratively updating the surrogate model based on observed function evaluations, BO intelligently selects the next evaluation point in order to balance exploration of promising regions of the search space with exploitation of regions likely to contain the global optimum. This balance is achieved through the acquisition function induced from the probabilistic surrogate model, which quantifies the utility of evaluating a particular point based on the current surrogate model (Shahriari et al. 2015; Jones et al. 1998; Frazier 2018).

In multi-agent systems, where subsystems are interconnected through shared variables or coupling constraints, standard Bayesian optimization (BO) approaches do not adequately address the interactions between subsystems. Furthermore, in such settings, the observations of each subsystem’s local objective function are often inaccessible to others, limiting the information available for conditioning the probabilistic surrogate models and inducing the acquisition functions. Consequently, while Bayesian optimization has gained traction for systems with unknown objectives, its application to multi-agent settings remains underdeveloped.

This paper addresses this research gap by presenting a general-purpose multi-agent Bayesian optimization framework that is compatible with a wide array of decomposition methods, with both centralized coordination and peer-to-peer coordination. Here each agent uses a probabilistic surrogate model for the local unknown objective function that is used to induce an acquisition function. The key idea of the proposed method is to append the local acquisition functions with additional coordinating terms. These terms, that are derived according to the different decomposition methods, are designed based on the information exchanged among agents within the shared environment, thereby enabling each subsystem to adapt its local BO decision-making process in response to the collective environment.

**Related work** Significant portion of research on BO has been devoted towards Batch BO to enable parallelizing the BO evaluations and expedite the optimization process. Here a single probabilistic surrogate model is used, and specifically designed acquisition functions are used to generate a batch of input queries, and each input query is evaluated in parallel (Contal et al. 2013; Desautels et al. 2014; Eriksson et al. 2019; Hernández-Lobato et al. 2017; Kandasamy et al. 2018). By evaluating multiple queries simultaneously, parallel BO can explore different regions of the search space in parallel, and all the parallel observations are pooled into a single dataset that is used to update the single GP model. In contrast to parallel or batch Bayesian optimization, where multiple candidate points are evaluated simultaneously within a single optimization process, multi-agent Bayesian optimization (MABO) involves the parallel execution of multiple independent BO processes, each representing a subsystem or agent. In MABO, each agent operates autonomously and conducts its own Bayesian optimization using only local data to optimize its local objective function, and coordinates with the other agents to reach the overall optimum, without sharing its local cost data. ADMM was recently used with BO in a framework called ADMM-BO by Ariafar et al. (2019). However, this was done under the context of handling unknown constraints for constrained BO, as opposed to multi-agent BO. van de Berg et al. (2023) considers a enterprise-wide optimization setting that is cast as a bi-level optimization problem, and a data-driven framework is adopted for the outer level coordinator to achieve consensus on the shared variables, while perfect knowledge of the local cost and the constraints for the different subsystems (inner level) are assumed. Recently, we proposed the first steps towards a multi-agent Bayesian optimization (MABO) using ADMM where the local cost is unknown (Krishnamoorthy and Paulson 2023), and empirically demonstrated the MABO framework for coordinated decision-making in multi-agent systems with affine coupling constraints.

**Main contribution**:  Building on our recent work (Krishnamoorthy and Paulson 2023), the main contribution of this paper is to formulate the problem as a general-purpose multi-agent Bayesian optimization framework that can be used with a wide range of decomposition methods, thus bridging advancements in BO and distributed optimization methodologies. Furthermore, this paper analyzes the regret bounds for the proposed general-purpose MABO framework with affine coupling constraints. To the author’s knowledge, regret analysis for a multi-agent BO framework, especially a general-purpose one applicable for a broad range of decomposition methods has not appeared before. We then discuss how the presented regret analysis can also extend to problems with unknown nonlinear coupling constraints for a class of engineering applications with real-time control capability. The proposed approach is then empirically demonstrated using four different classes of decomposition methods.

The reminder of the paper is organized as follows: Sect. 2 introduces different decomposition methods and establishes a unified framework with two key components: the local objective, which is unknown, and a coordinating function consisting of known elements. Section 3 presents the general-purpose multi-agent Bayesian optimization framework. Regret analysis for the proposed general purpose MABO is presented in Sect. 4. Section 5 discusses extension of the proposed framework to settings with black-box constraints. Numerical experiments for the different decomposition frameworks that demonstrate the proposed approach are presented in Sect. 6, before concluding the paper in Sect. 7.

**Notational remark** $\{(\cdot )_{i}\}$ by default denotes the set of variables $\{(\cdot )_{i}\}_{i=1}^N$ unless specified otherwise. $\Vert \cdot \Vert$ by default denotes the Euclidean norm. For a set ${\mathcal {C}}$, $|{\mathcal {C}}|$ denotes the cardinality of the set, and $\max (0,\cdot )$ is denoted by $[\;\cdot \;]^+$.

## Problem setup

### Global variable consensus problem

Consider the optimization problem,

1 $$\begin{aligned} \min _{x\in {\mathcal {X}}}\; \sum _{i=1}^Nf_i(x) \end{aligned}$$

where ${\mathcal {X}}$ is a simple set. This can be written with local variables $x_{i} \in {\mathcal {X}} \subseteq {\mathbb {R}}^n$ and a global variable $x_{0} \in {\mathcal {X}}$

2 $$\begin{aligned} \min _{x_{0},\{x_{i}\}\in {\mathcal {X}}}&\; \sum _{i=1}^Nf_i(x_{i})\nonumber \\ s.t.&\; x_{i} - x_{0} = 0 \; | \; \lambda _i, \quad i = 1,\dots ,N \end{aligned}$$

for which the augmented Lagrangian can be written as

3 $$\begin{aligned} {\mathcal {L}}_{\rho }({x_{0},\{x_{i}\},\{\lambda _{i}\}}):= \sum _{i=1}^N \left[ f_{i}(x_{i}) + \lambda _i^{{\textsf{T}}} (x_{i}-x_{0}) + \frac{\rho }{2}\Vert x_{i}-x_{0}\Vert ^2 \right] \end{aligned}$$

where $\lambda _i \in {\mathbb {R}}^n$ denotes the Lagrange multiplier of the consensus constraints. This global variable consensus problem can be decomposed and solved using ADMM (Boyd et al. 2011), given by the iterates

4a $$\begin{aligned} x_{0,k+1}&= \frac{1}{N}\sum _{i=1}^N\left[ x_{i,k} + \frac{\lambda _{i,k}}{\rho } \right] \end{aligned}$$

4b $$\begin{aligned} x_{i,k+1}&= \arg \min _{x_{i}\in {\mathcal {X}}}\; f_{i}(x_{i}) + \lambda _{i,k}^{{\textsf{T}}}x_{i} + \frac{\rho }{2}\Vert x_{i}-x_{0,k+1}\Vert ^2,&\forall i = 1,\dots ,N \end{aligned}$$

4c $$\begin{aligned} \lambda _{i,k+1}&= \lambda _{i,k} + \rho (x_{i,k+1} - x_{0,k+1}),&\forall i=1,\dots ,N \end{aligned}$$

Here each local agent is responsible for solving (4b) in parallel, and reports their local decisions $x_{i}$ to the central collector (4a).

### Decentralized consensus using peer-to-peer (P2P) coordination

Instead of using a central collector (4a) to achieve consensus, the *N* agents can coordinate over a network that can be represented by a undirected graph ${\mathcal {G}} = (\{1,\dots ,N\},{\mathcal {E}})$, where ${\mathcal {E}}:= \{(i,j)\}$ denotes the set of all edges. Existence of an edge (*i*, *j*) indicates that agent *i* communicates with agent *j*, and vice versa. The set ${\mathcal {C}}_{i}$ denotes the neighborhood of the $i^{th}$ agent, i.e., set of agents that directly communicate with agent *i*. The consensus problem (1) in this case can be decomposed by introducing copies of the primal variables $\{x_{i}\}$, denoted by $\{z_{i}\}$, resulting in the formulation

5 $$\begin{aligned} \min _{\{x_{i}\},\{z_{i}\}}&\sum _{i=1}^{N} f_{i}(x_{i}) \nonumber \\ s.t. \quad&x_{i} - z_{j} = 0 \; | \; \lambda _{ij} , \quad \forall (i,j) \in {\mathcal {E}} \nonumber \\&x_{i} - z_{i} = 0 \; | \; \lambda _{ii}, \quad \forall i= 1, \dots , N \end{aligned}$$

The augmented Lagrangian of (5) can then be written as

6 $$\begin{aligned} \min _{\{x_{i}\},\{z_{i}\}}&\sum _{i=1}^{N} \left[ f_{i}(x_{i}) + \sum _{j \in {\mathcal {C}}_{i}}\lambda _{ij}^{{\textsf{T}}} x_{i}+\lambda _{ii}^{{\textsf{T}}}x_{i} - \sum _{j \in {\mathcal {C}}_{i}}\lambda _{ij}^{{\textsf{T}}} z_{j}-\lambda _{ii}^{{\textsf{T}}}z_{i} + \frac{\rho }{2}\sum _{j \in {\mathcal {C}}_{i} \cup \{i\}}\Vert x_{i}-z_{j}\Vert ^2\right] \end{aligned}$$

This can be decomposed in an alternating directions fashion (Notarstefano et al. 2019), given by the iterations

7a $$\begin{aligned} x_{i,k+1}&= \arg \min _{x_{i}} f_{i}(x_{i})+ \sum _{j \in {\mathcal {C}}_{i}}\lambda _{ij,k}^{{\textsf{T}}} x_{i}+\lambda _{ii,k}^{{\textsf{T}}}x_{i} + \frac{\rho }{2}\sum _{j \in {\mathcal {C}}_{i}\cup \{i\}}\Vert x_{i}-z_{j,k}\Vert ^2, \; \nonumber \\&\quad \forall i= 1, \dots , N \end{aligned}$$

7b $$\begin{aligned} z_{i,k+1}&= \arg \min _{z_{i}} -\sum _{j \in {\mathcal {C}}_{i}}\lambda _{ji,k}^{{\textsf{T}}} z_{i}-\lambda _{ii,k}^{{\textsf{T}}}z_{i} + \frac{\rho }{2}\sum _{j \in {\mathcal {C}}_{i}\cup \{i\}}\Vert x_{j,k+1}-z_{i}\Vert ^2, \; \nonumber \\&\quad \forall i= 1, \dots , N \end{aligned}$$

7c $$\begin{aligned} \lambda _{ij,k+1}&= \lambda _{ij,k} + \rho (x_{i,k+1}-z_{j,k+1}), \; \lambda _{ii,k+1} = \lambda _{ii}^k + \rho (x_{i,k+1}-z_{i,k+1}), \nonumber \\&\quad \forall j \in {\mathcal {C}}_{i}, \; i = 1, \dots ,N \end{aligned}$$

Note that (7b) is an unconstrained QP, and thus has a closed-form solution given by,

8 $$\begin{aligned} z_{i,k+1} = \frac{1}{|{\mathcal {C}}_{i}|+1} \sum _{j \in {\mathcal {C}}_{i} \cup \{i\}} x_{j,k+1} + \frac{1}{\rho (|{\mathcal {C}}_{i}|+1)} \left( \sum _{j \in {\mathcal {C}}_{i}}\lambda _{ji,k}^{{\textsf{T}}} + \lambda _{ii,k}^{{\textsf{T}}} \right) \end{aligned}$$

Here each local agent is responsible for solving (7a) in parallel.

### Resource allocation

Consider the optimal resource allocation problem of the form

9 $$\begin{aligned} \min _{\{x_{i} \in {\mathcal {X}}_{i}\}} \; \sum _{i=1}^{N}f_{i}(x_{i}) \qquad s.t. \; \sum _{i=1}^{N}A_{i}x_{i} \le b \end{aligned}$$

For which the Lagrangian is given by relaxing the resource constraint,

10 $$\begin{aligned} \min _{\{x_{i} \in {\mathcal {X}}_{i}\}} \; \sum _{i=1}^{N}f_{i}(x_{i}) + \lambda ^{{\textsf{T}}}\left( \sum _{i=1}^{N}A_{i}x_{i} - b\right) \end{aligned}$$

Since the Lagrangian is additively separable, this can can be decomposed as

11a $$\begin{aligned} x_{i,k+1}&= \arg \min _{x_{i} \in {\mathcal {X}}_{i}} f_{i}(x_{i})+ \lambda _{k}^{{\textsf{T}}}A_{i}x_{i},&\forall i= 1, \dots , N \end{aligned}$$

11b $$\begin{aligned} \lambda _{k+1}&= \left[ \lambda _{k} + \rho \left( \sum _{i=1}^N A_{i}x_{i,k+1} - b\right) \right] ^+ \end{aligned}$$

resulting in the Lagrangian decomposition framework. Here each local agent is responsible for solving (11a) in parallel.

Of course the problem above can also be solved using ADMM by using the augmented Lagrangian. For a specific class of problems with $$ A_{i} = I$$, $$ b= 0 $$ and an equality coupling constraint (which is known as optimal exchange problem)

12 $$\begin{aligned} \min _{\{x_{i} \in {\mathcal {X}}_{i}\}} \; \sum _{i=1}^{N}f_{i}(x_{i}) \qquad s.t. \; \sum _{i=1}^{N}x_{i} = 0 \end{aligned}$$

The problem can be decomposed using the following ADMM iterates (Boyd et al. 2011, Sec. 7.3.2)

13a $$\begin{aligned} x_{i,k+1}&= \arg \min _{x_{i} \in {\mathcal {X}}_{i}} f_{i}(x_{i})+ \lambda _{k}^{{\textsf{T}}}x_{i} + \frac{\rho }{2}\Vert x_{i} - x_{i,k} + {x}_{0,k}\Vert ^2,&\forall i= 1, \dots , N \end{aligned}$$

13b $$\begin{aligned} \lambda _{k+1}&= \lambda _{k} + \rho {x}_{0,k+1} \end{aligned}$$

where ${x}_{0,k} = \frac{1}{N}\sum _{i=1}^N x_{i,k}$. Here the Lagrange multiplier $\lambda \in {\mathbb {R}}^n$ acts as the shadow price of the shared resource, which is adjusted to balance the supply and demand in this micro market setting (Boyd et al. 2011; Stojanovski et al. 2015).

## General-purpose multi-agent Bayesian optimization

To this end, the subproblems that each agent solves locally in the different decomposition frameworks (4b), (7a), (11a), (13a) can be expressed using a common abstraction of the form

14 $$\begin{aligned} x_{i,k+1} = \arg \min _{x_{i} \in {\mathcal {X}}_{i}} f_{i}(x_{i}) + \delta _i(x_{i},p_{i,k}) \end{aligned}$$

where $f_{i}(x_{i})$ denotes the self-interest of the agent, $x_{i} \in {\mathcal {X}}_{i} \subseteq {\mathbb {R}}^n$, $\delta _i: {\mathbb {R}}^n \times {\mathbb {R}}^{n_{p}} \rightarrow {\mathbb {R}}$ denotes the coordinating terms, and the parameter $p_{i,k} \in {\mathbb {R}}^{n_{p}}$ denotes the information that an agent receives from the shared environment (either from the central coordinator or connected neighbors). For the different classes of problems described above, $\delta _i(x_{i},p_{i,k})$ and $p_{i,k}$ are detailed in Table 1.

**Table 1:** The coordinating terms and the parameter for the three different problem classes considered above

(4b)	$$ p_{i,k}:= [x_{0,k+1},\lambda _{i,k}]^{\textsf{T}}$$
	$$\delta _{i}(x_i,p_{i,k}):= \lambda _{i,k}^{{\textsf{T}}}x_{i} + \frac{\rho }{2}\Vert x_{i}-x_{0,k+1}\Vert ^2 $$
(7a)	$$ p_{i,k}:= [\{\lambda _{ij,k},z_{j,k}\}_{j\in {\mathcal {C}}_{i} \cup \{i\}}]^{{\textsf{T}}} $$
	$$ \delta _{i}(x_i,p_{i,k}):= \sum _{j \in {\mathcal {C}}_{i}}\lambda _{ij,k}^{{\textsf{T}}} x_{i}+\lambda _{ii,k}^{{\textsf{T}}}x_{i} + \frac{\rho }{2}\sum _{j \in {\mathcal {C}}_{i}\cup \{i\}}\Vert x_{i}-z_{j,k}\Vert ^2$$
(11a)	$$ p_{i,k}:= \lambda _{k}$$
	$$ \delta _{i}(x_i,p_{i,k}):= \lambda _{k}^{{\textsf{T}}}A_{i}x_{i} $$
(13a)	$$ p_{i,k}:=[x_{i,k}, {x}_{0,k}, \lambda _{k}]^{{\textsf{T}}}$$
	$$ \delta _{i}(x_i,p_{i,k}):= \lambda _{k}^{{\textsf{T}}}x_{i} + \frac{\rho }{2}\Vert x_{i} - x_{i,k} + {x}_{0,k}\Vert ^2 $$

### Assumption 1

The local cost function $f_{i}(x_{i})$ are unknown, but can be evaluated at any $x_{i} \in {\mathcal {X}}_{i} \subset {\mathbb {R}}^n$ to obtain noisy observations of the local objective function

15 $$\begin{aligned} y_{i,t} = f_{i}(x_{i,t}) + \nu _{i,t}, \quad \forall i = 1,\dots ,N \end{aligned}$$

where $$ \nu _{i,t} \sim {\mathcal {N}}(0, \tau ^2I)$$.

In the different problem classes considered above, the coordinating term $\delta _{i}(x_{i},p_{i,k})$ is known, and the local objective function $f_{i}(x_{i})$ is the only unknown part.

### Assumption 2

The data set ${\mathcal {D}}_{i}^t=\{X_{i,t},Y_{i,t}\}$ cannot be shared across the subsystems, i.e., subsystem *i* cannot access data from any other subsystem $j \ne i$, where $X_{i,t}:= [x_{i,1},x_{i,2},\dots ,x_{i,t}]$ and $Y_{i,t}:= [y_{i,1},y_{i,2},\dots ,y_{i,t}]$ are a sequence of sample points and the corresponding cost observations up to step *t*.

### Assumption 3

For all $$ i= 1,\dots ,N $$, $$ f_{i} \in {\mathbb {H}}_{k_{i}} $$ where $$ k_{i}: {\mathbb {R}}^n \times {\mathbb {R}}^n \rightarrow {\mathbb {R}} $$ are kernel function, and $$ {\mathbb {H}}_{k_{i}} $$ are their corresponding reproducing kernel Hilbert spaces (RKHS). Furthermore, $$ f_{i} $$ have a RKHS norm bounded by $$ B_{i} $$, i.e., $$ \Vert f_{i}\Vert _{{k_{i}} } \le B_{i} $$ for some $$ B_{i} >0$$.

Such a multi-agent black-box optimization problem under assumptions 1 and 2 can be solved by applying Bayesian optimization separately to each agent. Each subproblem uses a Gaussian process surrogate to capture the unknown local objective function

16 $$\begin{aligned} f_{i}(x_{i}) \sim {{\mathcal {G}}}{{\mathcal {P}}}(\mu _i(x_{i}),k_{i}(x_{i},x_{i}')), \quad \forall i = 1\dots ,N \end{aligned}$$

where $\mu _i(x_{i}) = {\mathbb {E}}[f_{i}(x_{i})]$ and $k_{i}(x_{i},x_{i}'):= {\mathbb {E}}\left[ (f_{i}(x_{i}) - \mu _i(x_{i}))(f_{i}(x_{i}')-\mu _i(x_{i}')) \right]$ are the mean and covariance functions, specified by

17a $$\begin{aligned} \mu _{i,t}(x_{i})&= k_{i}(X_{i,t},x_{i})^{{\textsf{T}}}\left( K_{i,t} + \tau I \right) ^{-1} Y_{i,t} \end{aligned}$$

17b $$\begin{aligned} k_{i,t}(x_{i},x_{i}')&= k_{i}(x_{i},x_{i}') - k_{i}(X_{i,t},x_{i})^{{\textsf{T}}}\left( K_{i,t} + \tau I \right) ^{-1}k_{i}(X_{i,t},x_{i}') \end{aligned}$$

where $k_{i}(X_{i,t},x) = [k_{i}(x_{i,1},x_{i}),k_{i}(x_{i,2},x_{i}),\dots ,k_{i}(x_{i,t},x_{i})]^{{\textsf{T}}}$, $K_{i,t} = [k_{i}(x_{i}, x_{i}')]_{x_{i},x_{i} \in X_{i,t}}$ is the $t\times t$ positive definite covariance matrix, and the posterior variance is given by $\sigma ^2_{i,t} (x_{i})= k_{i,t}(x_{i},x_{i})$.

At time step *t*, the unknown local objective is replaced by any suitable acquisition function $\alpha _{i,t}: {\mathbb {R}}^n \rightarrow {\mathbb {R}}$ induced from the Gaussian process surrogate conditioned on the local data set ${\mathcal {D}}_{i}^{t-1}$. However computing the next action for each local agent by optimizing only the acquisition function $x_{i,t} = \arg \min _{x_{i} \in {\mathcal {X}}_{i}} \alpha _{i,t}(x_{i})$ as done in standard Bayesian optimization would lead to each agent taking independent decisions without any coordination. To enable coordination, this paper proposes to embed the local Bayesian optimization framework for each agent within the distributed optimization framework for a broad class of decomposition approaches. Using the common abstraction (14), each agent now solves

18 $$\begin{aligned} x_{i,t} = \arg \min _{\{x_{i} \in {\mathcal {X}}_{i}\}} \alpha _{i,t}(x_{i}) + \delta _i(x_{i},p_{i,k}) \end{aligned}$$

to compute the next action, where the coordinating terms $\delta _{i}(x_{i},p_{i,k})$ and the parameters $p_{i,k}$ are chosen according to their corresponding decomposition framework used, as detailed in Table 1. The subproblems solve the local BO problem (18) for a given parameter $p_{i,k}$ that are updated outside the BO loop, more generally denoted as

19 $$\begin{aligned} p_{i,k+1} = {\mathcal {H}}_{i}(p_{i,k},\{x_{i,k}\}), \quad \forall i=1,\dots ,N \end{aligned}$$

where the operator ${\mathcal {H}}_{i}$ is given by (4a) and (4c) for the problem setup in Sect. 2.1, (7b) and (7c) for the problem setup in Sect. 2.2, and (11b) and (13b) for the problem setup in Sect. 2.3, respectively.

**Fig. 1:**
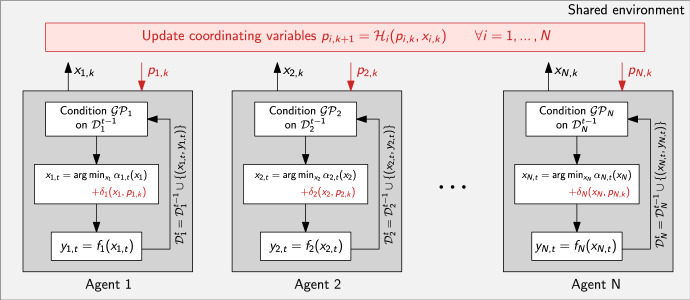
Schematic representation of the general purpose MABO framework, where each agent uses a local BO loop

**Algorithm 1:**
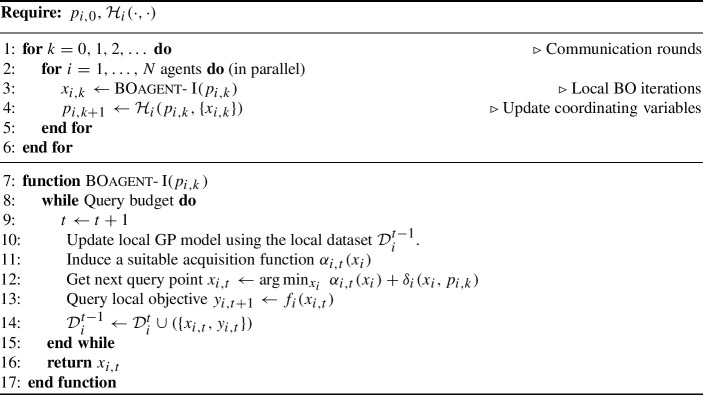
General-purpose Multi-agent Bayesian Optimization

### Remark 1

(*Data privacy*) The Gaussian Process (GP) model in each subsystem is conditioned solely on its local cost measurement, eliminating the need to share local data with other subsystems, thereby satisfying assumption 2. This feature enables a group of agents to coordinate their actions effectively without sharing their local data with one another.

### Remark 2

(*Hierarchical structure of the BO iterations and decomposition iterations*) The proposed generalized multi-agent Bayesian optimization framework exhibits a hierarchical structure characterized by two distinct layers of iterations. These involve local Bayesian optimization iterations within each subproblem, denoted by $$ t=1,2,\dots $$, and the distributed optimization iterations required for coordination among the agents, denoted by $$ k =0,1,2,\dots $$ (i.e., the communication rounds). Notably, the generalized MABO approach provides the flexibility to implement multiple Bayesian optimization iterations $$ t=1,2,\dots $$ per communication round *k*. For instance, following each communication round, the local agents can execute one or several Bayesian optimization iterates specific to the communicated information $$ p_{i,k} $$. In the case of multiple BO iterations per communication round, the latest BO iterate $$x_{i,t} = x_{i,k} $$ is used in the parameter update operation () (cf. Algorithm 1). Additionally, the different subproblems can conduct a different number of Bayesian optimization iterations between each communication round. This feature proves advantageous when dealing with varying local evaluation times and budgets among the different agents.

### Remark 3

(*Choice of acquisition function*) It is essential to highlight that the choice of the local acquisition function $\alpha _{i,t}(x_{i})$ is completely independent of the coordinating term $\delta _{i}(x_{i},p_{i,t})$. This means that any acquisition function from Bayesian Optimization (BO) literature can seamlessly integrate with the proposed Multi-Agent Bayesian Optimization (MABO) approach. Additionally, there is flexibility in choosing different acquisition functions for each subsystem within the multi-agent network as demonstrated by Krishnamoorthy and Paulson (2023). In simple terms, each agent has the freedom to select its own local acquisition function without impacting the overall structure of the MABO strategy.

## Regret analysis

This paper has thus far introduced a general-purpose decomposable Bayesian optimization (BO) framework for multi-agent systems that satisfies Assumptions 1–3, where coordination terms $\delta _i(x_{i},p_{i,k})$ are incorporated into the local acquisition functions $\alpha _{i,t}(x_{i})$ (cf. (18)). A critical question that now arises is how the inclusion of these coordination terms $\delta _i\left( x_i,p_{i,k}\right)$ affects the regret arising from the black-box nature of the subsystem objectives. This section is devoted to addressing this question.

### Definition 1

(Maximum information gain) For each agent $i=1,\dots ,N$, the maximum information gain at time *t* is defined as

20 $$\begin{aligned} \gamma _{i,t}:= \max _{X_{i,t}\in {\mathcal {X}}_{i}; |X_{i,t}|=t}\; {\textbf{I}}(Y_{i,t},f_{i}) \end{aligned}$$

which quantifies the reduction in uncertainty about $f_{i}$ after observing $Y_{i,t}$. For a Gaussian process, ${\textbf{I}}(Y_{i,t},f_{i}) = \frac{1}{2}\log |I + \tau ^{-1}K_{i,X_{i,t}}|$, where $K_{i,X_{i,t}} = [k_{i}(x_{i},x_{i}')]_{x_{i},x_{i}'\in X_{i,t}}$

### Assumption 4

(*Well-calibrated GP*) For each agent $i=1,\dots ,N$, the GP models are well-calibrated, such that w.p. at least $1-\omega$,

$$\begin{aligned} |\mu _{i,t}(x_{i}) - f_{i}(x_{i}) | \le \beta _{i,t+1}^{1/2}\sigma _{i,t}(x_{i}), \; \forall x_{i} \in {\mathcal {X}}_{i},\; \forall t >0 \end{aligned}$$

This assumption essentially means that the true function is contained within the confidence intervals with high probability. As shown in Chowdhury et al. (2017), this assumption can be satisfied by choosing $\beta _{i,t+1}^{1/2} = B_{i} + \tau \sqrt{2(\gamma _{i,t} +1 + \ln (1/\omega ))}$. Defining the upper and lower confidence bounds for each unknown local objective as

21a $$\begin{aligned} l_{i,t}(x_{i})&:= \mu _{i,t-1}(x_{i}) - \beta _{i,t}^{1/2} \sigma _{i,t-1}(x_{i}) \end{aligned}$$

21b $$\begin{aligned} u_{i,t}(x_{i})&:= \mu _{i,t-1}(x_{i}) + \beta _{i,t}^{1/2} \sigma _{i,t-1}(x_{i}) \end{aligned}$$

Assumption 4 implies that $$ f_{i}(x_{i}) \in [l_{i,t}(x_{i}),u_{i,t}(x_{i})] $$ for all $$ x_{i} \in {\mathcal {X}}_{i} $$, for all $$ i=1,\dots ,N $$ and $$ t >0 $$ w.p. $$ 1-\omega $$. In a black-box setting, regret quantifies the difference between the value of the best decision we could have made with perfect information of the local objective functions, and the decisions we actually make at a given time *t*. As such, we define regret for a given parameter $$ p_{i,k}$$ that the local BO agent receives from the shared environment, since this helps quantify and analyze the regret arising from the black-box nature of the local objective functions.

22 $$\begin{aligned} r_{i,t}:= \left( f_{i}(x_{i,t}) + \delta _{i}(x_{i,t},p_{i,k}) \right) - \left( f_{i}(x_{i}^*) + \delta _{i}(x_{i}^*,p_{i,k}) \right) , \quad \forall i= 1,\dots ,N \end{aligned}$$

In the multi-agent Bayesian optimization setting with additive cost, we define regret of the overall system as follows

23 $$\begin{aligned} r_{t}:= \sum _{i=1}^{N} \left( f_{i}(x_{i,t}) + \delta _{i}(x_{i,t},p_{i,k}) \right) - \sum _{i=1}^{N} \left( f_{i}(x_{i}^*) + \delta _{i}(x_{i}^*,p_{i,k}) \right) \end{aligned}$$

and cumulative regret as

24 $$\begin{aligned} R_{T}:= \sum _{t=1}^{T} r_{t} \end{aligned}$$

### Lemma 1

(Lemma 4 (Chowdhury et al. 2017)) For a series of points $x_{i,1}, x_{i,2}, \dots , x_{i,T}$ selected by the local Bayesian optimization algorithm by agent *i*, we have

25 $$\begin{aligned} \sum _{t=1}^T \sigma _{i,t-1}(x_{i,t}) \le 2\sqrt{(T+2)\gamma _{i,T}} \end{aligned}$$

### Theorem 1

Under assumptions 3 and 4, by using $\alpha _{i,t}(x_{i}) = l_{i,t}(x_{i})$ for all $i=1,\dots ,N$, w.p. at least $1- \omega$, for all $t>1$, and for all $p_{i,k}$

26 $$\begin{aligned} r_{t} \le 2\sum _{i=1}^{N} \beta _{i,t}^{1/2} \sigma _{i,t-1}(x_{i,t}) \end{aligned}$$

### Proof

$$\begin{aligned} r_{t}&= \sum _{i=1}^{N} \left( f_{i}(x_{i,t}) + \delta _{i}(x_{i,t},p_{i,k}) - f_{i}(x_{i}^*) - \delta _{i}(x_{i}^*,p_{i,k}) \right) \\&= \sum _{i=1}^{N} \left( f_{i}(x_{i,t}) - l_{i,t}(x_{i,t}) + l_{i,t}(x_{i,t})+ \delta _{i}(x_{i,t},p_{i,k}) - f_{i}(x_{i}^*) - \delta _{i}(x_{i}^*,p_{i,k}) \right) \\&\le \sum _{i=1}^{N} \left( u_{i,t}(x_{i,t}) - l_{i,t}(x_{i,t}) + l_{i,t}(x_{i,t})+ \delta _{i}(x_{i,t},p_{i,k}) - l_{i,t}(x_{i}^*) - \delta _{i}(x_{i}^*,p_{i,k}) \right) \\&=\sum _{i=1}^{N} \left( u_{i,t}(x_{i,t}) - l_{i,t}(x_{i,t})\right) + \sum _{i=1}^{N} \left( l_{i,t}(x_{i,t})+ \delta _{i}(x_{i,t},p_{i,k}) - l_{i,t}(x_{i}^*) - \delta _{i}(x_{i}^*,p_{i,k}) \right) \\&\le \sum _{i=1}^{N} \left( u_{i,t}(x_{i,t}) - l_{i,t}(x_{i,t})\right) = 2\sum _{i=1}^{N} \beta _{i,t}^{1/2} \sigma _{i,t-1}(x_{i,t}) \end{aligned}$$

where the last inequality follows from the fact that

$$\begin{aligned} l_{i,t}(x_{i,t})+ \delta _{i}(x_{i,t},p_{i,k}) \le l_{i,t}(x_{i}^*) + \delta _{i}(x_{i}^*,p_{i,k}), \quad \forall i=1,\dots ,N \end{aligned}$$

since $$x_{i,t} = \arg \min _{x_{i} \in {\mathcal {X}}_{i}}\; l_{i,t}(x_{i}) + \delta _{i}(x_{i},p_{i,k})$$ $$\square $$

### Theorem 2

Under the same setup as Theorem 1, the cumulative regret of the generalized multi-agent Bayesian optimization is bounded, given by

27 $$\begin{aligned} R_{T} \le 4 \sum _{i=1}^N \beta _{i,T}^{1/2}\sqrt{(T+2)\gamma _{i,T}} \end{aligned}$$

### Proof

$$\begin{aligned} R_{T}&= \sum _{t=1}^{T} r_{t} \le 2\sum _{t=1}^{T}\sum _{i=1}^{N} \beta _{i,t}^{1/2} \sigma _{i,t-1}(x_{i,t}) \le 2\sum _{i=1}^{N} \beta _{i,T}^{1/2}\sum _{t=1}^{T} \sigma _{i,t-1}(x_{i,t}) \\&\le 4\sum _{i=1}^{N} \beta _{i,T}^{1/2}\sqrt{(T+2)\gamma _{i,T}} \end{aligned}$$

$$\square $$

The regret analysis provides three main insights into the generalized MABO framework. Firstly, and more importantly, the regret due to the unknown local objective is independent of the coordinating terms $\delta _{i}(x_{i},p_{i})$, and hence is independent of the decomposition method used. Secondly, the regret of an agent is not directly dependent on the actions of the other agent, whereby an agent can carry out as many BO iterations per communication round for a given $p_{i,k}$ to reduce its local regret (cf. Remark 2). However, note that $p_{i,k}$ depends on the actions of the other agents. Finally, the total regret of the overall multi-agent system is simply the sum of the individual regret of the agents. As the agents acquire more information through local function evaluations, the uncertainty about the unknown local objective decreases, and the information gain diminishes irrespective of $p_{i,k}$ and the decomposition method used, i.e., the cumulative regret is bounded, and this bound is independent of the shared information $p_{i,k}$ and/or the number of communication rounds *k*. Simply put, the addition of the coordinating terms $\delta _{i}(x_{i},p_{i,k})$ does not complicate the regret bound of the local BO agents.

To this end we establish that the local BO agents do not incur any additional regret due to the multi-agent setting. On the question of regret for the $p_{i,k}$ update, note that the update rule ${\mathcal {H}}_{i}\left( \cdot \right)$ does not have any black-box terms, and hence does not incur additional regret. The actual convergence of $p_{i,k}$ will depend on the specific decomposition algorithm used and its corresponding update rule ${\mathcal {H}}_{i}\left( \cdot \right)$ along with its necessary assumptions as in any white-box distributed optimization.

## Black-box coupling constraints

So far, we considered affine coupling constraints in the different distributed optimization problem formulations, such that $\delta _{i}(x_{i},p_{i})$ did not contain any unknown black-box terms. Hence the coordinating terms did not require a black-box treatment. Consider now the optimization problem with unknown coupling constraints of the form

28a $$\begin{aligned} \min _{\{x_{i} \in {\mathcal {X}}_{i}\}} \;&\sum _{i=1}^{N}f_{i}(x_{i}) \end{aligned}$$

28b $$\begin{aligned} s.t. \;&\sum _{i=1}^{N}g_{i}(x_{i}) \le 0 \end{aligned}$$

where in addition to the local objective functions $f_{i}:{\mathbb {R}}^n \rightarrow {\mathbb {R}}$, the constraint terms $g_{i}:{\mathbb {R}}^n \rightarrow {\mathbb {R}}^m$ are also unknown, but can be measured. When using Lagrangian decomposition for this problem, the coordinating term $\delta _i (x_{i},p_{i}):= \lambda ^{{\textsf{T}}}g_{i}(x_{i})$ with $p_{i} = \lambda$. One approach to handle this is to place individual Gaussian process surrogate models for each unknown constraint $g_{i}(x_{i})$. Note that this would involve placing *m* GP surrogates in each subsystem under the assumption that the *m* elements of the constraint vector $g_{i}(x_{i})$ are uncorrelated. As shown in Zhang et al. (2023), such an assumption may be acceptable in most cases. Xu et al. (2023b) studies this approach, where $g_{i}(x_{i})$ in $\delta _i (x_{i},p_{i})$ is replaced with the lower confidence bound ${\underline{g}}_{i} (x_{i})$ in order to provide regret bounds (under the assumption that $g_{i}(x_{i}) \in [{\underline{g}}_{i} (x_{i}), {\bar{g}}_{i} (x_{i}) ]$ with high probability. However, the cumulative regret and violation bounds are only valid under the case where Lagrange multiplier $\lambda$ is updated using the lower confidence bound ${\underline{g}}_{i} (x_{i})$ as opposed to the actual constraint measurement $g_{i}(x_{i})$, despite the actual measurement being available after each evaluation. This also implies that the choice of the GP hyperparameters affect the decomposition framework.

**Fig. 2:**
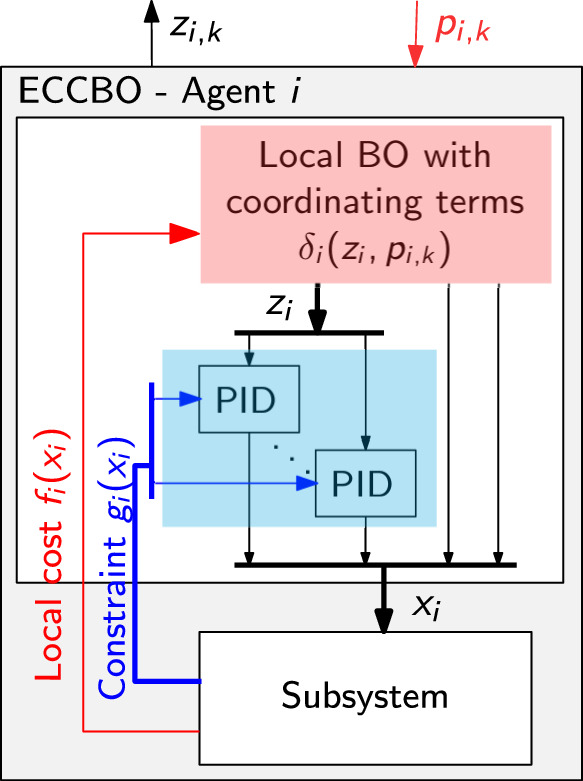
Schematic representation showing the transformed problem using the embedded constraint control (ECCBO) formulation for subsystem *i*

For a class of engineering systems capable of being controlled in real-time, an alternative approach can be devised using Bayesian optimization with embedded constraint control (ECCBO), that was recently proposed by Krishnamoorthy (2024). ECCBO exploits the vertical decomposition with time scale separation between the optimization and the control layer (Skogestad 2023). Here $g_{i}(x_{i})$ is controlled to some setpoint $z_{i}$ using $x_{i}$ as the degrees of freedom in the faster control layer, and the setpoint $z_{i}$ is optimized by the slower Bayesian optimization layer above. I.e., the degrees of freedom for the BO are transformed from $x_{i}$ to $z_{i}$. ECCBO avoids the need for any surrogate models for the unknown constraints $g_{i}(x_{i})$, thereby ensuring zero cumulative constraint violation (at steady-state) without the need for any additional GPs and their associated assumptions. Here, the key idea is to transform the original problem as

29a $$\begin{aligned} \min _{\{x_{i} \in {\mathcal {X}}_{i}, z_{i} \}} \;&\sum _{i=1}^{N}f_{i}(x_{i}) \end{aligned}$$

29b $$\begin{aligned} s.t. \;&g_{i}(x_{i}) =z_{i}, \quad \forall i = 1,\dots ,N \end{aligned}$$

29c $$\begin{aligned}&\sum _{i=1}^{N}z_{i} \le 0 \end{aligned}$$

Since $g_{i}(x_{i})$ measurement is locally available in each subsystem, the equality constraint (29b) within each subsystem can be satisfied using a feedback controller, where $x_{i}$ is used as the manipulated variable to control the unknown constraint $g_{i}$ to a setpoint of $z_{i}$ as shown in Fig. 2. Controlling a constraint using feedback controller, such as a PI controller, does not require a model, making it compatible with the black-box framework. By using the ECCBO formulation in each subsystem, the coupling constraints become affine in the transformed variables $z_{i}$. As such, the regret analysis presented in Sect. 4 applies to the case with nonlinear unknown constrains using the embedded constraint control formulation ECCBO. Detailed description of ECCBO can be found in (Krishnamoorthy 2024).

### Remark 4

In addition to black-box coupling constraints, subsystems may have black-box local constraints that require tailored handling approaches, as studied in the BO literature. Earlier works on constrained BO (Gardner et al. 2014; Gelbart et al. 2014) addressed feasibility probabilistically at the end of the query budget but lacked guarantees on cumulative regret and constraint violations, even in single-agent settings (Xu et al. 2023a). Safe Bayesian Optimization (Sui et al. 2015; Krishnamoorthy and Doyle III 2022) ensures zero constraint violations through a conservative approach but provides no bounds on cumulative regret (Xu et al. 2023a). Penalty-Based Methods, such as CONFIG (Xu et al. 2023a), achieve bounded guarantees for both regret and constraint violations, aligning directly with the framework in this paper. Thus, the regret analysis here applies seamlessly when CONFIG handles unknown local constraints. Finally, embedded constraint control approaches like ECCBO (Krishnamoorthy 2024) transform constrained BO into equivalent unconstrained problems for systems with real-time control capabilities, preserving the regret guarantees established in this paper for handling unknown local constraints.

## Illustrative examples

This section demonstrates the use of the general purpose multi-agent Bayesian optimization with four classes of decomposition methods described in Sects. 2.1–2.3, applied to two engineering examples, namely, a vehicle platooning problem, and an optimal resource allocation in a subsea oil production network. In all the examples considered below, the proposed MABO framework was implemented in Python using the GPy package (GPy 2012) to model the unknown local cost function $f_{i}(x_{i})$ in each subsystem. The different subsystems use GP-LCB for the local acquisition function, i.e., $\alpha _i(x_{i}) = l_i(x_{i})$. The local optimization problem (18) with $\delta _{i}(x_{i},p_{i,k})$ and $p_{i,k}$ chosen appropriately (cf. Table 1) is optimized using the L-BFGS method using the SciPy package (Virtanen et al. 2020).

### Example 1a—vehicle platooning with a centralized coordinator

Optimal cruising speeds of trucks are pivotal in minimizing fuel consumption per vehicle kilometer (i.e fuel economy), particularly in vehicle platooning scenarios. In such setups, where a fleet of heavy-duty vehicles travels closely in a single-file formation on highways, selecting the appropriate cruising speeds becomes crucial. A recent experimental study conducted by Lammert et al. (2014) highlighted this importance, revealing that while fuel economy improved across all platooning speeds, certain speeds were found to offer the best overall fuel efficiency (Lammert et al. 2014). This research underscores the significance of optimizing cruising speeds to maximize fuel efficiency in truck platooning applications, emphasizing the need to identify the most effective speed ranges for optimal performance. However, the impact of cruising speed on fuel consumption for an individual vehicle is not uniform and varies based on factors such as cargo load, road conditions, time since last engine maintenance, etc. Consequently, creating precise models capable of determining the optimal platoon speed is a challenging task.

**Fig. 3:**
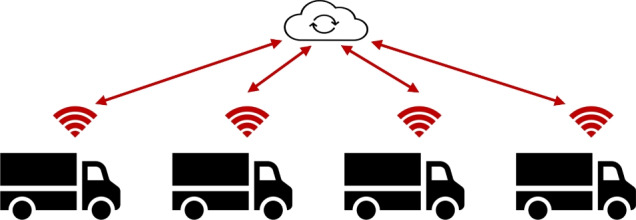
Example 1a: Schematic representation of the vehicle platooning problem with centralized coordination

**Fig. 4:**
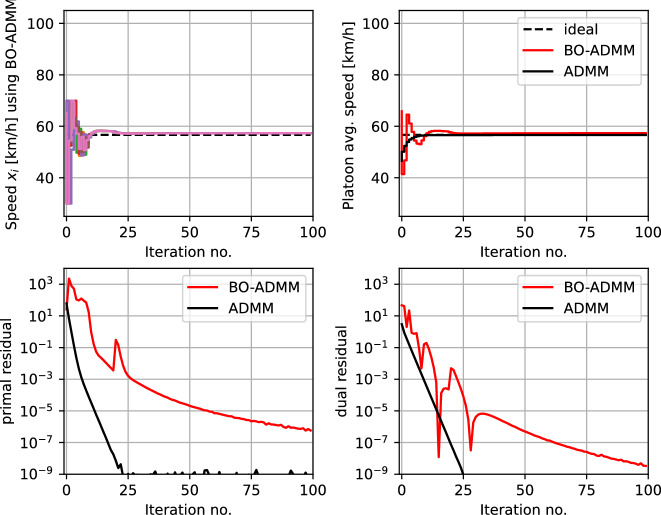
Example 1a—Vehicle platooning problem with consensus ADMM: simulation results using the proposed MABO framework used together with consensus ADMM, benchmarked against model-based ADMM

Black-box formulations prove valuable in the context of vehicle platooning, especially when the platoon comprises diverse vehicles with distinct configurations or from different manufacturers. The challenge arises in coordinating the different vehicles to minimize overall fuel consumption of the platoon because each vehicle may have a unique optimal speed w.r.t. fuel economy. Such problems can be formulated as a global variable consensus problem of the form (1), and solved using ADMM (cf. 4a–4c).

We consider a platoon of $N= 7$ vehicles that coordinate to find the optimal cruising speed of the platoon that minimizes the overall fuel consumption. The fuel consumption in [g/veh-km] of the $i^{th}$ vehicle is simulated using the model derived by Sobrino et al. (2016), where

$$\begin{aligned} f_{i}(x_{i}) = a_{i} + \frac{b_{i}}{x_{i}} + c_{i}x_{i} + d_{i}x_{i}^2 \end{aligned}$$

where $x_{i}$ is the vehicle speed in [km/h]. The nominal model parameters, as experimentally calibrated and documented (Sobrino et al. 2016, Section 5), serve as the baseline. For simulating $N = 7$ distinct vehicles (i.e., the zeroth order oracles), we introduce perturbations by randomly sampling model parameters from a uniform distribution, varying within ±20% of the nominal values from Sobrino et al. (2016). Each agent uses a GP model with RBF kernel (length scale = 10, and variance = 100) with bias. GP-LCB acquisition function is used in the local BO loops with $\beta _{i,t} = 4$.

Figure 4 shows the simulation results using a black-box ADMM formulation with $\rho = 0.1$. The top subplot shows the speed of the individual vehicles $x_{i,t}$ that uses local BO loops to find the optimal speed. The coordinating terms $\delta _{i}(x_{i},p_{i,k})$ (as shown in Table 1) enables the vehicles to coordinate to reach consensus on the speed of the platoon. Since the local objective is unknown, the vehicles initially explore different speeds $x_{i } \in [30,70]$ km/h, and as the uncertainty about the local objective decreases, the vehicles reach consensus. This is also seen in the average speed of the platoon $x_{0,t}$ shown in the top right subplot (in red). This is also benchmarked against the traditional model-based ADMM with perfect knowledge of the local objective function (i.e., the white box approach) in each vehicle (shown in black). The bottom subplots show the primal and dual residuals using our proposed black-box ADMM (in red) compared with the model-based ADMM (in black), from which we can conclude that the MABO algorithm using ADMM framework is effectively minimizing the overall objective function and satisfies the coupling constraints.

### Example 1b—Dynamic platooning with time-varying network

**Fig. 5:**
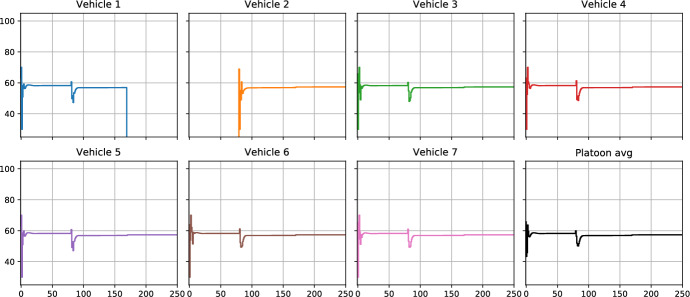
Example 1b—individual vehicle speeds with time-varying platoon using the proposed MABO framework that shows the addition of vehicle 2 and departure of vehicle 1

**Fig. 6:**
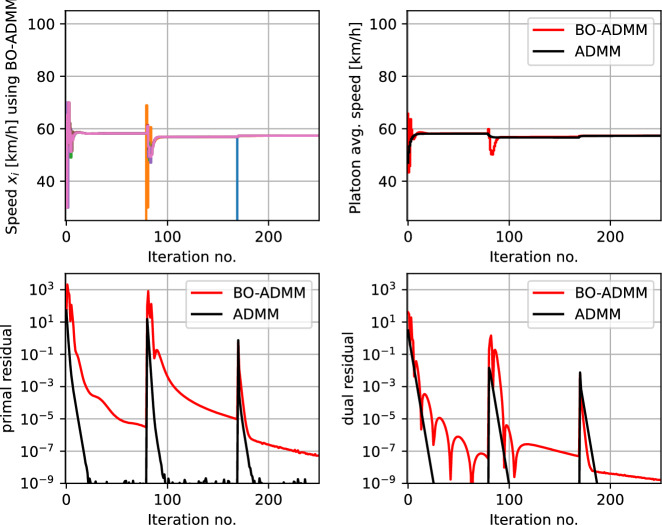
Example 1b—dynamic platooning problem with time-varying platoon: simulation results using the proposed MABO framework used together with consensus ADMM, benchmarked against model-based ADMM

To illustrate the flexibility and robustness of the proposed general-purpose Bayesian Optimization (BO) framework, we consider a dynamic vehicle platooning scenario where the number of vehicles in the platoon changes over time. The simulation begins with six vehicles (vehicles 1, 3, 4, 5, 6, and 7) using local BO to determine their optimal cruising speeds while coordinating to achieve consensus within the platoon. As shown in Figs. 5 and 6, the vehicles initially explore different cruising speeds and gradually gain information about their local objectives, leading to a reduction in regret and convergence to a common cruising speed.

At iteration $k= 80$, vehicle 2 joins the platoon, increasing the total number of vehicles to seven. Vehicle 2 starts exploring its local objective using BO, as seen by its trajectory (orange curve in Fig. 5). The other six vehicles, having already gained sufficient information about their local objectives, do not engage in additional exploration (cf. individual vehicle speeds plotted in Fig. 5). Instead, they adjust their speeds based on the coordinating terms $p_{i,k}$ to accommodate the new vehicle. This demonstrates the framework’s adaptability: the introduction of vehicle 2 does not adversely affect the regret of other vehicles, as their previously acquired information remains valid. Since the overall regret of the network is a sum of the local regrets, as the regret for vehicle 2 diminishes with more information gain, the overall regret of the platoon also diminishes after the addition of vehicle 2.

At iteration $k=170$, vehicle 1 leaves the platoon. The remaining six vehicles quickly coordinate to determine the new optimal cruising speed without the need for additional exploration, leveraging their previously acquired information. The ability to adjust seamlessly to such changes highlights the robustness of the proposed framework.

The proposed BO framework is benchmarked against the traditional model-based ADMM approach, which assumes perfect knowledge of the local objectives (white-box method). The comparison is shown in Fig. 6 in terms of the average platoon speed, as well as, primal and dual residuals, where the red curves represent the proposed BO-based framework (MABO), and the black curves represent the model-based ADMM approach. This dynamic platooning example demonstrates the proposed framework’s ability to handle time-varying systems efficiently while maintaining robust performance and coordination among subsystems.

### Example 1c—Vehicle platooning with decentralized peer-to-peer coordination

**Fig. 7:**
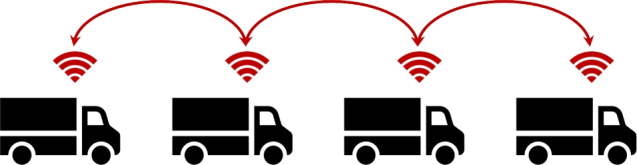
Example 1c: schematic representation of the vehicle platooning problem with decentralized peer-to-peer coordination

**Fig. 8:**
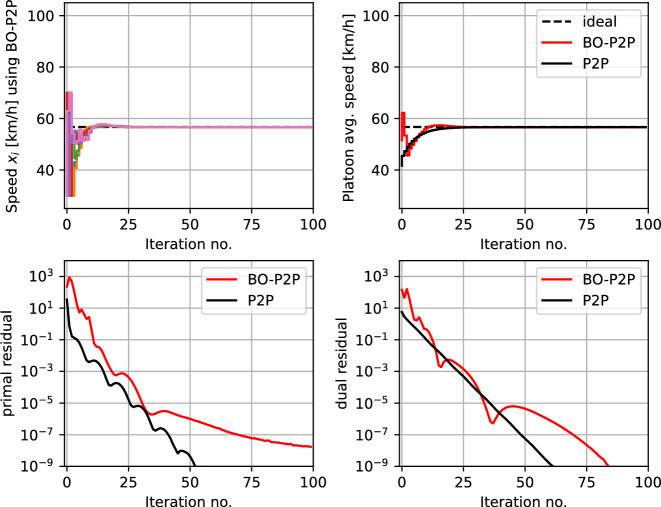
Example 1c—Vehicle platooning problem with decentralized peer-to-peer (P2P) coordination: simulation results using the proposed MABO framework used together with decentralized peer-to-peer coordination, benchamrked against model-based decentralized consensus problem

We consider the same problem as in Sect. 6.1, but instead of using a centralized coordinator, the vehicles coordinate in a peer-to-peer (P2P) fashion, to achieve consensus on the speed of the platoon. Here, each vehicle only communicates with the closest neighbor, leading to a chain communication network as shown in Fig. 7. This problem can be formulated as (5), and solved iteratively as described in Sect. 2.2. The zeroth order oracle, as well as the GP models used by the different vehicles are the same as in Sect. 6.1.

Figure 8 shows the simulation results using a black-box P2P formulation with $\rho = 0.1$. The top subplot shows the speed of the individual vehicles $x_{i,t}$ that uses local BO loops to find the optimal speed. The coordinating terms $\delta _{i}(x_{i},p_{i,k})$ (as shown in Table 1) enables the vehicles to coordinate to reach consensus on the speed of the platoon via peer-to-peer coordination. Since the local objective is unknown, the vehicles initially explore different speeds, and as the uncertainty about the local objective decreases, the vehicles reach consensus. This is also seen in the average speed of the platoon shown in the top right subplot (in red). This is benchmarked against the model-based decentralized consensus problem with perfect knowledge of the local objective function in each vehicle (shown in black). The bottom subplots shows the primal and dual residuals using our proposed black-box framework (in red) compared with the model-based framework (in black), from which we can conclude that the MABO algorithm using the peer-to-peer coordination framework is effectively minimizing the overall objective function and satisfies the coupling constraints.

### Example 2a—Optimal resource allocation in offshore oil production using Lagrangian decomposition

**Fig. 9:**
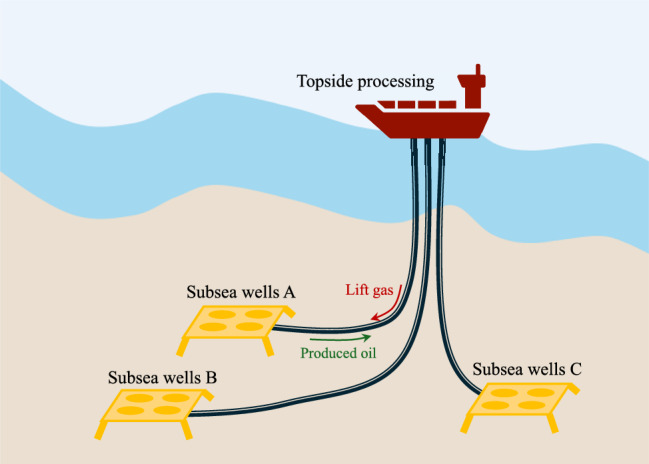
Example 2: schematic representation of the subsea oil production system with a shared processing facility

In this example, we consider the problem of offshore gas-lifted oil production from subsea wells. Often subsea wells tapping into remote reservoirs are linked to a shared processing facility due to the potential lack of economic viability in building separate topside processing facilities, particularly for reservoirs with relatively low recoverable resources. It is common for wells drawing from various reservoir sections to be operated by different companies, while utilizing the same topside processing facility (cf. Fig. 9). Given the limited resources on offshore platforms, optimal allocation of resources such as compressed gas used for gas-lift is essential for maximizing production. Utilizing distributed real-time production optimization facilitates optimal resource allocation in such production networks.

Detailed models are essential for effective production optimization, yet obtaining these models for complex reservoirs and varied well conditions can pose significant challenges. The availability of such detailed models may be limited, warranting the need for black-box optimization. Specifically in the case of gas-lifted wells, the effect of the lift gas $x_{i}$ on the oil produced from each well $w_{po,i}$ may be unknown. In this section, we formulate the optimization problem of optimizing the local objectives using Bayesian optimization, subject to the optimal resource allocation constraint, that couples the different subsystems together. Here we consider 3 subsea wells and 1 topside processing facility. The lift gas is compressed in the topside facilities, that is then distributed to the different wells to boost the oil production. The maximum available lift gas is limited to $x_{gl}^{\max } = 8$kg/s. This limited shared resource must then be optimally allocated to the different wells to maximize the total oil production.

This can be formulated in the form of (9) with $$ N=3 $$ subsystems, $$f_{i}(x_{i}) = w_{po,i} (x_{i})+ 0.1x_{i} $$ and $$ A_{i} = I $$ for $$ i=1,2,3 $$, and $$ b = x_{gl}^{\max } $$ and solved using Lagrangian decomposition as shown in (11).To simulate the subsea oil production network, we model each well as a differential algebraic equation (DAE) model with 3 differential states, 12 algebraic states, and 1 control input for each subsystem. The detailed model of the gas lifted wells can be found in Krishnamoorthy et al. (2019, 2018). Note that this DAE model is used as the zeroth order oracle when testing the multi-agent Bayesian optimization approach, and no model information is used by the MABO framework. The local BO loops use RBF kernel in the GP surrogates, and uses GP-LCB with $$\beta _{i,t} = 4$$. The proposed MABO is benchmarked against the standard model-based distributed optimization approach, that uses the same DAE model as the optimization model.

**Fig. 10:**
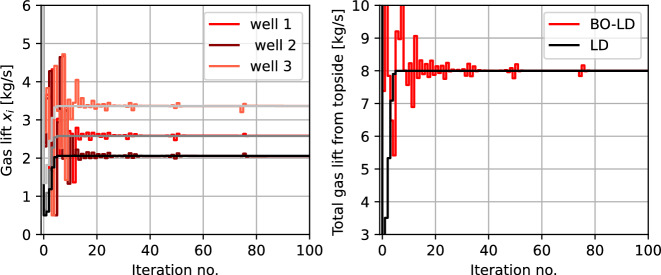
Example 2a—Optimal resource allocation in subsea production network using Lagrangian decomposition

Figure 10 shows the simulation results using a black-box Lagrangian decomposition formulation. The left subplot shows the gas lift rate $x_{i,t}$ for the three wells, that uses local BO loops. The total gas lift rate $\sum x_{i}$ is shown in the right subplot.The coordinating terms $\delta _{i}(x_{i},p_{i,k}) = \lambda _{k}$ (which denotes the shadow price of the shared resource) enables optimal allocation of the limited resource. Since the local objective is unknown, different gas lift rates $x_{i} \in [0.5,5]$kg/s are explored initially, and as the uncertainty about the local objective decreases, optimal resource allocation is achieved. This is benchmarked against the model-based variant of the same problem with perfect knowledge of the local objective function in each subsystem (shown in black), from which we can conclude that the MABO algorithm using Lagrangian decomposition effectively minimizes the overall objective function and satisfies the coupling constraints.

**Fig. 11:**
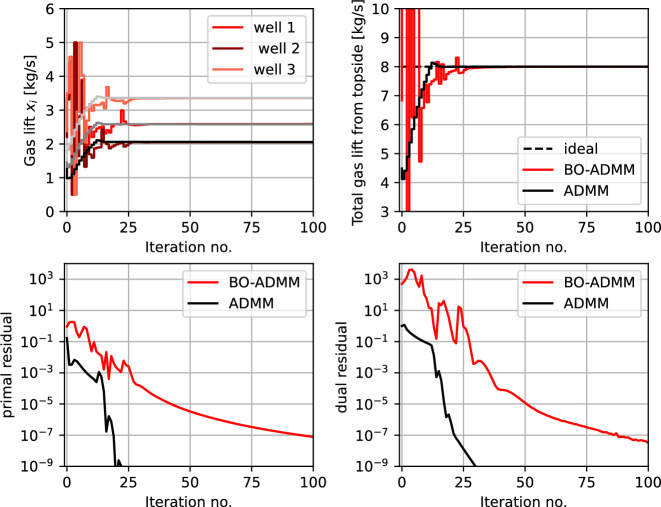
Example 2b—Optimal resource allocation in subsea production network using sharing ADMM

### Example 2b—Optimal resource allocation in offshore oil production using sharing ADMM

As described in Sect. 2.3, the optimal resource allocation problem can also be formulated as an optimal exchange problem and solved using ADMM. In this case, the same gas lift optimization problem can be formulated with $$ N= 4 $$ subsystems in the form of (13), where $$ i=1,2,3 $$ denotes the three subsea wells, and $$ i=4 $$ denotes the topside processing, where the decision-variable $$ x_{i} $$ denotes the lift gas that is allocated to each well for $$ i=1,2,3 $$ and $$ x_{4}$$ denotes the total amount of lift gas supplied from the topside process, such that $$ \sum _{i=1}^{4} x_{i}= 0 $$. This can be formulated in the form of (13), solved using ADMM as described above. Here $$f_{i}(x_{i}) = w_{po,i} (x_{i}) $$ for $$i = 1,2,3$$ and $$f_{4}(x_{4}) = - 0.1x_{4} $$. Subproblems $$i=1,2,3$$, uses local BO loops since the local cost is unknown, whereas $$f_{4}(x_{4}) = -0.1x_{4}$$ is known, and hence solves the local problem analytically, which is given as $$x_{4}^{k+1} = (0.1-\lambda _k)/\rho + x_{4}^k -x_{0}^k$$, where $$x_{0}^k = 0.25\sum _{i=1}^4x_{i}$$.

Figure 11 shows the simulation results using a black-box ADMM formulation with $\rho = 10$. The top left subplot shows the gas lift rate $x_{i,t}$ for the three wells, that uses local BO loops. The total gas lift rate $-x_{4}$ is shown in the top right subplot.The coordinating terms $\delta _{i}(x_{i},p_{i,k})$ (as shown in Table 1) enables optimal allocation of the limited resource. Since the local objective is unknown, different gas lift rates are explored initially, and as the uncertainty about the local objective decreases, optimal resource allocation is achieved. This is benchmarked against the model-based variant of the same problem with perfect knowledge of the local objective function in each subsystem (shown in black). The bottom subplots shows the primal and dual residuals using our proposed black-box framework (in red) compared with the model-based framework (in black), from which we can conclude that the MABO algorithm using the ADMM for the optimal exchange formulation effectively minimizes the overall objective function and satisfies the coupling constraints.

## Conclusion

In conclusion, this paper introduces a general-purpose approach to tackle the multi-agent Bayesian optimization (MABO) problem, wherein agents are interconnected via shared variables or constraints, each possessing local cost functions of unknown nature. Our proposed framework, compatible with various decomposition methods, extends traditional Bayesian optimization (BO) acquisition functions by incorporating coordinating terms (cf. Table 1) to foster coordination among black-box subsystems without necessitating the exchange of local data. Through rigorous analysis, Theorems 1 and 2 demonstrate that the regret of our general-purpose MABO remains independent of the coordinating terms, as well as the actions of the other agents. This adaptability to diverse decomposition methods ensures applicability across a wide array of distributed optimization algorithms. The regret analysis presented herein extends to cases with nonlinear unknown coupling constraints using the embedded constraint control formulation (ECCBO) for systems featuring real-time control capabilities.

Our numerical experiments corroborate the efficacy of the proposed MABO framework, highlighting its ability to navigate distributed optimization settings with unknown local objective. In all the simulation results presented in this work, the GP-LCB acquisition function is used for $\alpha _{i,t}(x_{i})$ for all subsystems *i*. However, note that our approach is not limited to the GP-LCB acquisition function, and one can use any acquisition function from the Bayesian optimization literature, see for e.g., Frazier (2018). In fact, one can also use different acquisition functions for different agents, as demonstrated in our earlier work (Krishnamoorthy and Paulson 2023). Conditioning the Gaussian process surrogate requires inverting the covariance matrix (cf. (17)), whose computational complexity increases with the dimensionality of the input space, hindering the widespread application of Bayesian Optimization (BO) in higher-dimensional settings. The proposed approach can also be used to decompose higher dimensional problems into smaller subproblems with local GPs, that can then be coordinated to achieve overall optimal solution. To this end, this paper contributes to bridging advancements in Bayesian optimization with distributed optimization methodologies, offering a versatile solution applicable to a broad spectrum of distributed optimization problems.

## Data Availability

The source code to reproduce the results can be made available upon reasonable request.
